# Characterization and distribution of HIV-infected cells in semen

**DOI:** 10.1080/22221751.2022.2049982

**Published:** 2022-03-21

**Authors:** Lin Gao, Yan-Mei Jiao, Ping Ma, Lijun Sun, Hongxin Zhao, An-Liang Guo, Xing Fan, Chao Zhang, Jin-Wen Song, Ji-Yuan Zhang, Fengmin Lu, Fu-Sheng Wang

**Affiliations:** a Department of Microbiology & Infectious Disease Center, School of Basic Medical Science, Peking University Health Science Center, Beijing, People’s Republic of China; b Senior Department of Infectious Diseases, The Fifth Medical Center of Chinese PLA General Hospital, National Clinical Research Center for Infectious Diseases, Beijing, People’s Republic of China; c Peking University 302 Clinical Medical School, Beijing, People’s Republic of China; d Nankai University Second People's Hospital, School of Medicine, Nankai University, Tianjin, People’s Republic of China; e Center for Infectious Diseases, Beijing Youan Hospital, Capital Medical University, Beijing, People’s Republic of China; f Clinical and Research Center of Infectious Diseases, Beijing Ditan Hospital, Capital Medical University, Beijing, People’s Republic of China; g Department of Immunology, School of Basic Medical Sciences, Cheeloo College of Medicine, Shandong University, Jinan, People’s Republic of China.

**Keywords:** HIV, spermatozoa, CD4^+^ T cell, CC chemokine receptor 5, CXC chemokine receptor 4

## Abstract

Semen is a known vector for both human immunodeficiency virus (HIV) infection and transmission. However, the distribution and characteristics of HIV-infected cells in semen remain unclear. Investigating the possibility of transmission through the spermatozoon in semen is of great clinical significance to improve the strategies for exposure prevention and assisted reproduction for HIV-infected partners. Twenty-six HIV-infected patients, including twelve treatment-naïve (TN) patients and fourteen antiretroviral treated (ART) patients, were enrolled in this study. HIV p24 protein in spermatozoa was detected using imaging flow cytometry and immunohistochemistry, and HIV RNA was identified using next-generation RNAscope *in situ* hybridization. Additionally, we described the rates of HIV-positive spermatozoon and CD4^+^ T lymphocytes in semen, and found that p24^+^ spermatozoon were mainly CD4 negative regardless of whether the patients received ART. Of note, p24-positive cells in semen are predominantly spermatozoa, and we confirmed that motile spermatozoa carried HIV into peripheral blood mononuclear cells of healthy men *in vitro*. Our findings provide evidence regarding the risk of HIV-infected spermatozoa.

## Highlights

- The number of HIV-positive spermatozoa was far greater than that of CD4^+^ T lymphocytes in the semen.
- p24 positive rates of CD4^+^ T lymphocytes in the semen were higher than spermatozoa.
- The HIV-positive rates of spermatozoa and CD4^+^ T lymphocytes in the semen were reduced significantly after ART.

## Introduction

Human immunodeficiency virus (HIV) infection is a major global public health challenge. Sexual transmission is the main route of HIV infection, and the reproductive system is involved in infection, replication, and transmission [1, 2]. The relevant cellular mechanisms are important for understanding HIV latency and, prevention and treatment strategies. However, there is an ongoing debate on whether HIV can infect the spermatozoon. Initially, HIV antigens were isolated from the lymphocytes in the semen of infected patients. Therefore, researchers believe that the antigens mainly originate from CD4^+^ T lymphocytes and macrophages in semen [3–5]. Some studies have shown that “sperm-washing” could result in the birth of seronegative children, believing that HIV only adheres to the surface of the spermatozoon [6–8]. Conversely, electron microscopy observations showed that HIV can enter the spermatozoon rather than merely adhering to the surface [9, 10]. Additionally, some studies reported the presence of HIV nucleic acids including RNA and/or proviral DNA in spermatozoa [11–13]. Recently, Domini et al. [14] found that human testicular germ cells (TGCs) could support early replication and integration of HIV *in vitro*, but this was not observed in ejaculated semen cells, including spermatozoa and immune cells. Studies on HIV-positive rates of spermatozoa and CD4^+^ T lymphocytes in the semen are still lacking. Primary receptors CD4 and co-receptor CC chemokine receptor 5 (CCR5) or CXC chemokine receptor 4 (CXCR4) are essential molecules for HIV entry into target cells [15]. Some studies suggested that HIV entry into germ cells may be achieved through a CD4-independent pathway [16, 17]. However, there are few reports on levels of these molecules in HIV-positive spermatozoa.

Semen is a viscous fluid, and ordinary washing methods cannot separate the cells from mucus; thus, semen cells cannot be grouped using conventional flow cytometry. Further, conventional detection methods cannot detect whether cells in semen are HIV positive. Morphological characteristics of a cell, such as its size, are used to distinguish spermatozoon and immune cells on imaging flow cytometry and next-generation RNAscope *in situ* hybridization. This helps avoid cell clustering and staining of mucus in the semen [18]. Highly sensitive next-generation RNAscope *in situ* hybridization with sixty targets binding to specific RNAs will show a positive signal only when both pol and gag probes are successfully identified, overcoming the low sensitivity of non-specific hybridization [19–21].

Herein, we aimed to explore the distribution characteristics of HIV in semen cells, including spermatozoon and CD4^+^ T cells, and to clarify whether HIV can enter the spermatozoon. We investigated whether HIV entry into spermatozoon was associated with the expression of receptor CD4 and co-receptors CCR5 or CXCR4. Additionally, we analyzed the relationship between the p24 positive rates of spermatozoon and disease progression and clarified infectiousness of spermatozoa that carried HIV.

## Materials and methods

### Study design and subjects

Twelve HIV-infected treatment-naïve patients (TN group, plasma HIV RNA >1,000 copies/mL without antiretroviral treatment), fourteen patients under long-term effective antiretroviral therapy (ART group, receiving antiretroviral treatment for more than 2 years with undetectable plasma HIV RNA viral load), and three healthy men were enrolled in this study (Supplementary Fig. 1). Before semen collection, all participants had a 48h-72h sexual abstinence period [22, 23], no opportunistic infections or cancer, and no hepatitis B or C virus coinfection. Baseline clinical characteristics including age, CD4^+^ T cell count, and plasma HIV RNA load are shown in Table 1. Paired blood and semen samples were provided voluntarily by participants at the Red Ribbon Home of the Fifth Medical Center of Chinese PLA General Hospital.

**Table 1.** Clinical characteristics of study population.

Group	Patient	Age (year)	Baseline HIV RNA (log10 copies/mL)	CD4^+^ T cell count (cells/μL)		HIV genotype	ART regimen	Duration of ART (years)
				Baseline	After ART			
TN (n=12)	P 1	23	4.76	261	−	B	−	−
	P 2	28	5.37	208	−	AE	−	−
	P 3	26	4.22	709	−	AE	−	−
	P 4	32	5.18	306	−	AE	−	−
	P 5	31	4.97	107	−	B	−	−
	P 6	22	2.42	818	−	B	−	−
	P 7	23	5.20	327	−	AE	−	−
	P 8	34	4.98	160	−	B	−	−
	P 9	33	4.49	420	−	B	−	−
	P 10	37	3.22	879	−	AE	−	−
	P 11	27	4.36	544	−	AE	−	−
	P 12	26	4.19	577	−	AE	−	−
ART (n=14)	P 13	31	4.52	283	516	B	DTG/TAF/FTC	8.67
	P 14	34	5.28	190	483	B	DTG/TAF/FTC	5.17
	P 15	24	5.68	210	589	B	DTG/TAF/FTC	7.42
	P 16	29	4.43	361	605	AE	3TC+TDF+LPv/r	3.67
	P 17	26	4.31	331	812	B	3TC+TDF+LPv/r	3.08
	P 18	28	5.55	157	535	AE	DTG/TAF/FTC	4.92
	P 19	26	4.23	484	500	AE	DTG/TAF/FTC	6.75
	P 20	26	4.04	254	447	AE	3TC+TDF+EFV	5.75
	P 21	24	5.10	178	411	B	3TC+TDF+EFV	3.08
	P 22	39	3.62	513	583	AE	3TC+TDF+EFV	3.75
	P 23	32	2.57	618	632	AE	DTG/TAF/FTC	4.11
	P 24	41	3.87	484	417	AE	DTG/TAF/FTC	2.51
	P 25	28	4.23	597	683	AE	3TC+TDF+EFV	4.24
	P 26	32	4.01	429	426	B	3TC+TDF+EFV	6.13
HC (n=3)	HC 1	30	−	741	−	−	−	−
	HC 2	28	−	702	−	−	−	−
	HC 3	23	−	789	−	−	−	−

HIV, human immunodeficiency virus; ART, antiretroviral therapy; TN, treatment-naïve; HC, health control; DTG, dolutegravir; TAF, tenofovir alafenamide fumarate; FTC, emtricitabine; 3TC, lamivudine; TDF, tenofovir dipivoxil fumarate; LPv/r, lopinavir/ritonavir.

### Sample preparation

Seminal fluid samples were obtained on self-masturbation and ejaculation in a sterile container. All samples were processed within 2 h of collection. Fresh semen samples were centrifuged at 2,000 rpm for 10 min. The cells were washed twice with fresh phosphate-buffered saline solution, filtered (40 μm), and subsequently used for flow staining. Fresh semen cells were fixed with 4% paraformaldehyde after smear preparation and stored at −20°C.

### Flow cytometry

Peripheral blood mononuclear cells (PBMCs) were isolated from fresh heparinized blood via Ficoll–Hypaque density gradient centrifugation (Pharmacia, Uppsala, Sweden). Multicolor flow cytometry with fluorescent conjugated antibodies obtained from BioLegend (San Diego, USA) were as follows: CD3-APC-Cy7 (clone SK7), CD8-Percp/Cyanine5.5 (clone SK1), CD56-BV421 (clone HCD56), CCR5-FITC (clone J418F1), and CXCR4-APC (clone RG5). CD4-APC-H7 (clone RPA-T4) and CD4-BV605 (clone RPA-T4) were obtained from BD Biosciences (New Jersey, USA). PBMCs were first stained at 4°C for 30 min with fluorescent antibodies under dark conditions. Cells were then permeabilized using a Cytofix/Cytoperm Kit (BD Bioscience) and stained with p24-PE (clone KC57) or p24-FITC (clone KC57) from Beckman Coulter (Eurocenter S.A, California, USA). The cells were fixed in 0.5% formaldehyde and analyzed using a FACSCanto^TM^ flow cytometer (BD Biosciences). The data were analyzed using the FlowJo software (TreeStar).

### Imaging flow cytometry

Semen cells were first stained with fluorescent antibodies such as CD3-APC-Cy7 (BioLegend, USA, clone SK7), CD4-BV605(BD, USA, clone RPA-T4), CD8-Percp/Cyanine5.5 (BioLegend, USA, clone SK1), CD56-BV421 (BioLegend, USA, clone HCD56), CCR5-FITC (BioLegend, USA, clone J418F1), CXCR4-APC (BioLegend, USA, clone RG5), p24-PE (Beckman Coulter, USA, clone KC57), and then analyzed using imaging flow cytometry.

The initial test of the staining plate contained all but one staining agent and fluorescence minus one control to determine the background staining of the channel. Cells were then acquired on an Amnis ImageStream Mk II flow cytometer (Luminex) using the INSPIRE 4.1 software with lasers set to maximum values without saturation in the brightest stains. Cell files (50,000) were collected with a cell classifier applied to the brightfield channel to capture a single-cell picture. Channels were as follows: Brightfield-Channel 1, FITC-Channel 2, PE-Channel 3, Percp/Cyanine5.5-Channel 5, BV421-Channel 7, BV605-Channel 10, APC-Channel 11, and APC/Cy7-Channel 12. Excitation lasers were used with the typical intensity settings of 405 nm (80 mW), 488 nm (100 mW), 594 nm (20 mW), and 658 nm (40 mW). All cell images were captured with the 40× objective and acquired at a rate of 200∼250 images per second. Data were analyzed using the IDEAS 6.2 (Amnis/EMDmillipore) software.

### Next-generation RNAscope in situ hybridization

HIV-1 clade B anti-sense probes (cat.317691) targeting the HIV gag-pol gene (507-4601), negative control probes (cat.310043), and positive control probes (cat.313901) were designed by Advanced Cell Diagnostics (Hayward, CA, USA). The RNAscope system was performed as described in previous studies [24, 25]. Briefly, after H_2_O_2_ treatment and protease digestion, semen cell smears were incubated for 2 h at 40°C with probes. The amplifiers and detection solutions in the RNAscope Multiplex Fluorescence Kit v2 reagent (cat. 323100) were sequentially added for hybridization signal amplification at the indicated times. The Aperio VERSA 8 Scanning System (Leica Microsystems, Wetzlar, Germany) was used for scanning and Aperio ImageScope (Leica Microsystems, Wetzlar, Germany) was used to obtain the images and count the cells.

### Immunohistochemistry

Semen smears were stained with hematoxylin–eosin to detect HIV p24 protein (Abcam, UK, cat. ab53841). Nuclei were stained light blue with hematoxylin. Images (100× and 400×) were acquired using an Olympus CX31 microscope and an Olympus FV1000 confocal microscope.

### HIV genotype testing

Pol and gag regions of DNA (QIAamp DNA Mini Kit [50], QIAGEN, cat. 51340) extracted from PBMCs of HIV-infected men were amplified and sent to Beijing Biomed Co., Ltd. for next-generation sequencing. HIV genotype results were collected after HIV sequence comparison in GenBank.

### Co-culture of spermatozoa and PBMCs of healthy men

Motile spermatozoa of HIV-infected men were generally collected using the “swim-up” method [26]; thus, we purified spermatozoon by the “swim-up” method (Supplementary Fig. 2) in co-culture experiments. CD8-positive cells were isolated from PBMCs of healthy men using CD8 microbeads (MACS, Germany, cat. 130-045-201); 1×10^6^ CD8-depleted PBMCs with purified 2×10^6^ spermatozoa were co-cultured in 2.5 mL of Biggers Whitten Whittingham (BWW) medium (Ygyr-Biotech, China, cat. LG2585) containing 20% sterile fetal bovine serum in a six-well plate for 7 days to verify the infectivity of HIV carried by the spermatozoa. Simultaneously, 1×10^6^ PBMCs with purified 2×10^6^ spermatozoa were co-cultured in the same system as described above. After 2 days, 1.25 mL medium was removed and 1.25 mL fresh BWW medium containing 20% fetal bovine serum and 0.1% interleukin-2 was added. Three days later, half of the medium was replaced with fresh medium, and the cells and supernatants were collected on day 7. Additionally, 2×10^6^ purified spermatozoa were cultured separately as a control group.

### HIV DNA detection

HIV RNA extracted from the cell culture supernatant (MagaBio plus Virus DNA/RNA Purification Kit, BioFlux) was reverse transcribed into cDNA. DNA was extracted from co-cultured cells using QIAamp® DNA MiniKit (QIAGEN, Germany, cat. 51304), and cDNA was used to perform nested polymerase chain reaction (PCR) using 2×*EasyTaq*® PCR SuperMix for PAGE (+dye) (TRAN, China, cat. AS112-11). The primer sequences used are listed in Supplementary Table 1. The reaction system and reaction conditions are listed in Supplementary Tables 2 and 3. PCR products were subjected to agarose gel electrophoresis.

### Ethical approval

The study subjects provided informed consent in accordance with the Declaration of Helsinki, and the study was approved by the institutional review board of the Fifth Medical Center of Chinese PLA General Hospital (KY-2021-12-32-1).

### Statistical analyses

All data were analyzed using the GraphPad Prism software version 8.0. Statistical differences between the two groups were evaluated using the Mann Whitney U nonparametric test. Correlations between variables were analyzed using Pearson’s correlation coefficient (*r)*. Statistical significance was set at *P* <0.05.

## Results

### Detection of HIV p24 protein and HIV RNA in spermatozoon of HIV-infections

**Figure 1.**
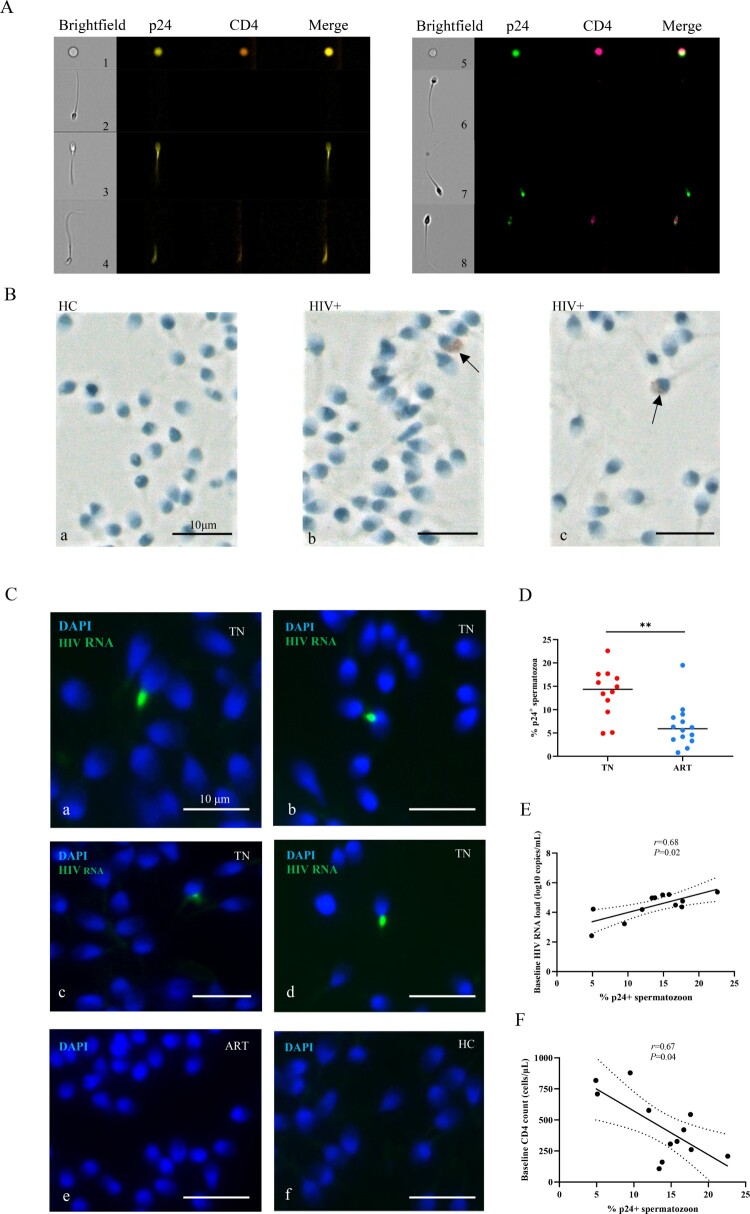
**Detection of HIV p24 protein and HIV RNA in spermatozoa.** (A) HIV p24 protein detected in semen cells from HIV-infected men by imaging flow cytometry. Left picture (p24-PE, CD4-BV605); right picture (p24-FTC, CD4-APC/Cy7). (B) HIV p24 detected in spermatozoon of HIV-infections in the TN group (b-c, black arrow) and healthy controls (a) via immunohistochemical analysis. The sperm nuclei are stained light blue with hematoxylin, and p24 Ab signal is red. (C) a–d, HIV RNA detected in spermatozoon from treatment-naïve (TN) HIV-infected patients using RNAscope; HIV RNA cannot be detected in spermatozoa from antiretroviral (ART) HIV-infected patients (e) and healthy men (f). Green fluorescence represents HIV RNA, and blue fluorescence represents nucleic acid in the sperm nucleus. (D) HIV p24-positive rates of spermatozoon in the TN and anti-retroviral therapy (ART) groups. (E–F) Linear regression between the ratio of p24^+^ spermatozoa and baseline plasma HIV RNA and baseline CD4^+^ T cell count. HIV p24-positive rates of spermatozoa were calculated from imaging flow cytometry cell counting. The Mann–Whitney U nonparametric test was used to compare the two groups. *P < 0.05. *r*, Pearson correlation coefficient. *P *< 0.05 is considered significant. The dotted line represents the 95% confidence interval.

As shown in Figure 1A, we detected p24^+^ spermatozoa in semen of the TN group via imaging flow cytometry (the fluorescence intensity of p24 is shown in Supplementary Table 4). p24^+^ spermatozoon were also detected in the semen smears of the TN group using an immunohistochemical assay (Figure 1B b-c), and no p24^+^ spermatozoa were identified in the semen smears of healthy men (Figure 1B a). RNAscope *in situ* hybridization was performed with the semen smears to further determine the presence of HIV in spermatozoa. Positive signals were detected in the TN group (Figure 1C, a–d), and no positive signal was detected in ART group (Figure 1C, e) and healthy men (Figure 1C, f). The p24-positive rates of spermatozoa were significantly reduced but were detectable after ART (Figure 1D). Moreover, the p24-positive rates of spermatozoa positively correlated with the plasma viral load (Figure 1E, *r  *= −0.68, *P* <0.05) and negatively correlated with CD4^+^ T cell count (Figure 1F, *r  *= 0.67, *P* <0.05). These results showed that spermatozoa from untreated HIV-infected individuals could act as carriers of HIV.

### Receptor CD4 and co-receptor CCR5 or CXCR4 levels of spermatozoon

**Figure 2.**
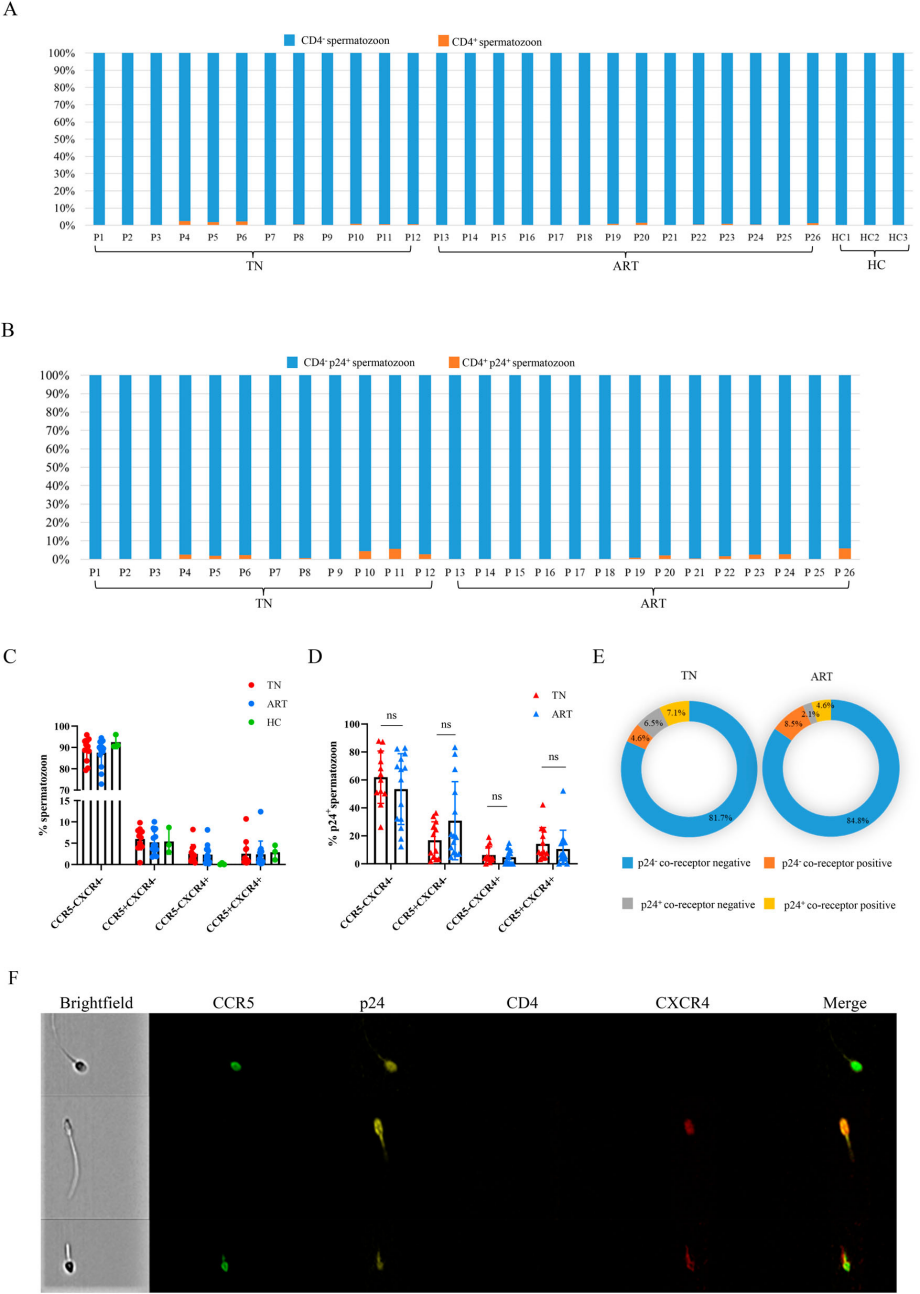
**Receptor CD4 and co-receptor CCR5 or CXCR4 levels of the spermatozoa.** (A) CD4 levels of spermatozoa in semen from HIV-infected men and healthy men. (B) CD4 levels of p24^+^ spermatozoa. (C) CCR5 or CXCR4 levels of spermatozoon from HIV-infected men and healthy men. (D) CCR5 or CXCR4 levels on p24^+^ spermatozoa from HIV-infected men. (E) CCR5 or CXCR4 levels of p24^-^ and p24^+^ spermatozoa in the TN and ART groups. (F) CCR5- or CXCR4-positive spermatozoa from HIV-infected men detected using imaging flow cytometry. CCR5-FITC; p24-PE; CD4-BV605; CXCR4-APC; CD3-APC/Cy7. The Mann–Whitney U nonparametric test was used to compare the two groups. ns, not significant.

CD4 is an essential receptor for HIV-infected cells. The overall expression level of CD4 on spermatozoon was very low (Figure 2A), and it was also found that p24^+^ spermatozoon were mainly CD4 negative (Figure 2B). In addition to the receptor CD4, CCR5 and CXCR4 are the two main co-receptors for HIV entry into target cells. We found a low level of CCR5 and CXCR4 in the spermatozoon and the expression levels of the patients varied greatly (Figure 2C). The proportion of CCR5 ^+ ^p24^+^ or CXCR4 ^+ ^p24^+^ spermatozoon was not significant different between the TN and ART groups (Figure 2D). CCR5 and CXCR4 levels of p24^-^ and p24^+^ spermatozoa were compared between the TN and ART groups (either CCR5 or CXCR4 positive was classified as the co-receptor positive group). We found that co-receptor levels of p24^+^ spermatozoa were higher than those of p24^-^spermatozoa in the TN and ART groups (Figure 2E). Typical flow cytometry images of CCR5 or CXCR4 positive spermatozoa in the TN group are shown in Figure 2F. The above results showed that spermatozoon carried HIV through a CD4-independent pathway and co-receptors appeared to contribute to it to some extent.

### CCR5 and CXCR4 Levels of CD4^+^ T lymphocytes in the semen

**Figure 3.**
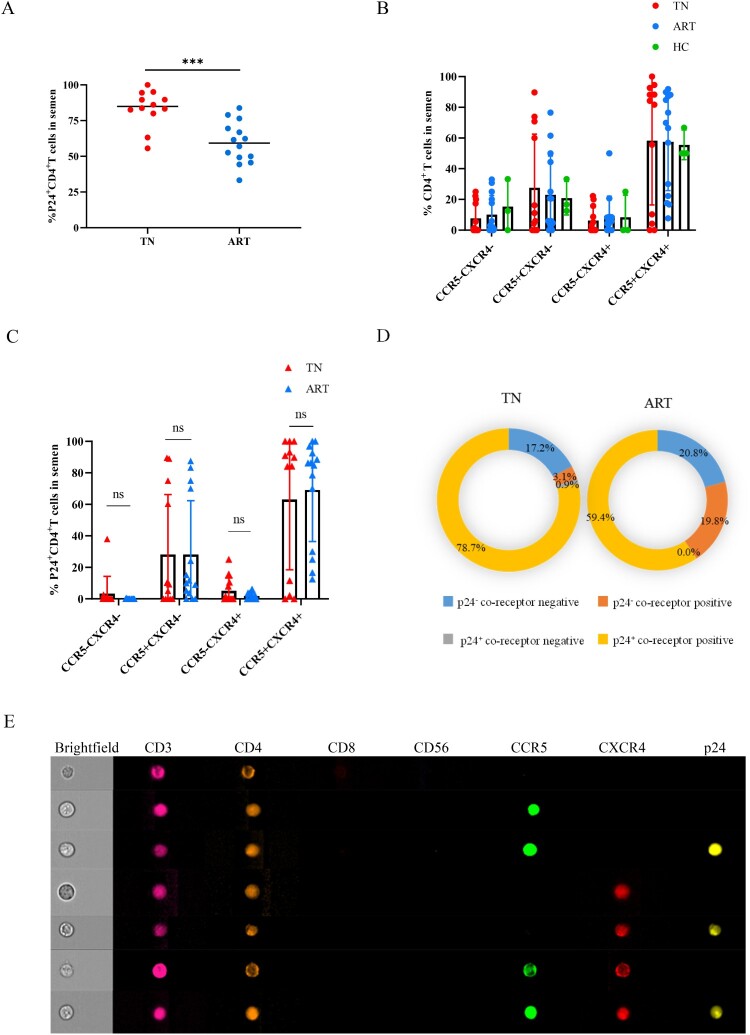
**CCR5 or CXCR4 levels on CD4^+^ T cells in semen.** (A) p24-positive rates of CD4^+^ T lymphocytes in semen from HIV-infected men in the TN and ART groups. (B) CCR5 or CXCR4 levels of CD4^+^ T cells in the semen of HIV-infected men and healthy men. (C) CCR5 or CXCR4 levels of p24^+^ CD4^+^ T cells in semen of the TN and ART groups. (D) CCR5 or CXCR4 levels of p24^-^CD4^+^ T cells and p24^+^ CD4^+^ T cells in semen of the TN and ART groups; either CCR5 or CXCR4 positive belongs to the co-receptor-positive group. (E) CD4^+^ T lymphocytes in semen detected on imaging flow cytometry. CCR5-FITC; p24-PE; CD8-Percp/Cy5.5; CD56-BV421; CD4-BV605; CXCR4-APC; CD3-APC/Cy7. The Mann–Whitney U nonparametric test was used to compare the two groups. *P < 0.05, **P < 0.01, ***P < 0.001. ns, not significant.

Furthermore, we analyzed the expression of the co-receptors CCR5 and CXCR4 on CD4 ^+ ^T lymphocytes in the semen. We found that the p24 positive rates of CD4 ^+ ^T lymphocytes significantly decreased after ART (Figure 3A), and the CCR5 and CXCR4 levels of CD4 ^+ ^T lymphocytes in semen (Figure 3B) and PBMCs (Supplementary Fig.3B) varied greatly among individuals. The proportions of CCR5 ^+ ^CXCR4^-^ or CCR5 ^+ ^CXCR4^+^ in p24^+^ CD4^+^ T lymphocytes in semen were not significantly different between the TN and ART groups (Figure 3C). The CCR5 or CXCR4 levels of p24^+^ CD4^+^ T lymphocytes in semen were significantly higher than those of p24^-^CD4^+^ T lymphocytes in the TN and ART groups (Figure 3D, either CCR5 or CXCR4 positive belongs to the co-receptor-positive group). Typical images of CD4^+^ T lymphocytes in semen are shown in Figure 3E. Effective ART could significantly reduce the p24-positive rates of CD4 ^+ ^T cells in the semen, and co-receptors were important for HIV infection.

### Proportion of p24^+^ cells in semen

**Figure 4.**
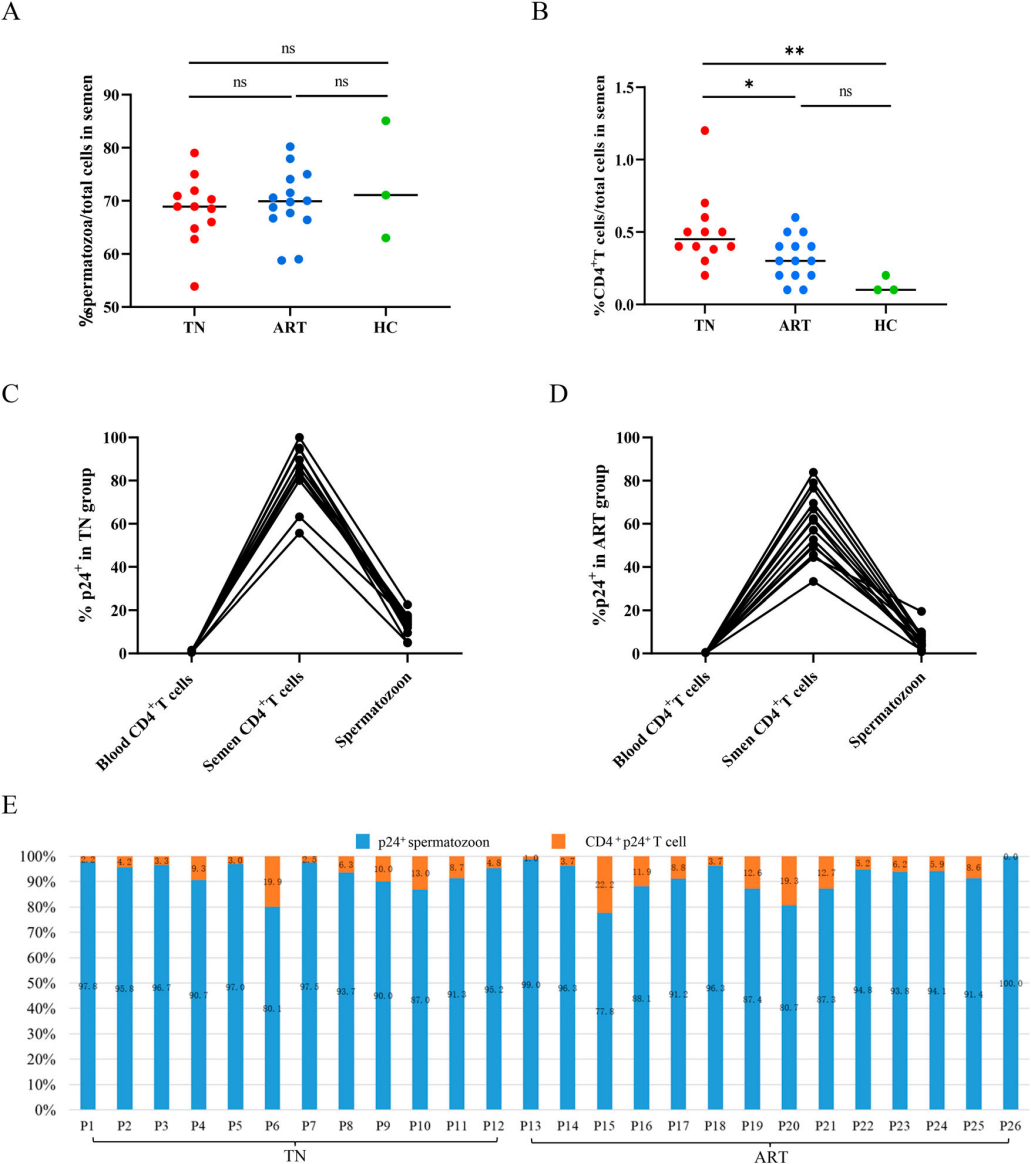
**Proportions of p24^+^ cells in semen.** Proportion of spermatozoa (A) and CD4^+^ T cells (B) in semen of HIV-infected men and healthy men. (C–D) Comparisons of p24-positive rates of spermatozoa and CD4^+^ T cells in the TN and ART groups. (E) Proportions of p24^+^ spermatozoa and p24^+^ CD4^+^ T lymphocytes of total p24 positive cells in semen. *P <0.05, **P <0.01, ***P <0.001. ns, not significant.

We further analyzed the proportion of p24^+^ cells in semen to determine whether the HIV-positive cells were predominantly CD4^+^ T cells. Spermatozoa were the main cells in semen of both healthy and HIV-infected individuals (Figure 4A). Although the proportion of CD4^+^ T cells of the TN group were significantly higher than that of healthy men, the overall proportion of CD4^+^ T cells in semen was still very low (Figure 4B). We further observed that CD4^+^ T lymphocytes in semen generally had higher p24-positive rates than spermatozoa and CD4^+^ T lymphocytes in peripheral blood, regardless of treatment (Figure 4C,D). Considering HIV p24-positive cells in semen, the proportion of p24-positive CD4^+^ T cells was approximately 6.3% (IQR 3.7%–11.4%) and the proportion of p24-positive spermatozoa was approximately 93.7% (IQR 88.6%–96.3%) (Figure 4E). Thus, the main HIV-carrying cells in semen were spermatozoon.

### Purified spermatozoon from HIV infections could infect PBMCs from healthy men

**Figure 5.**
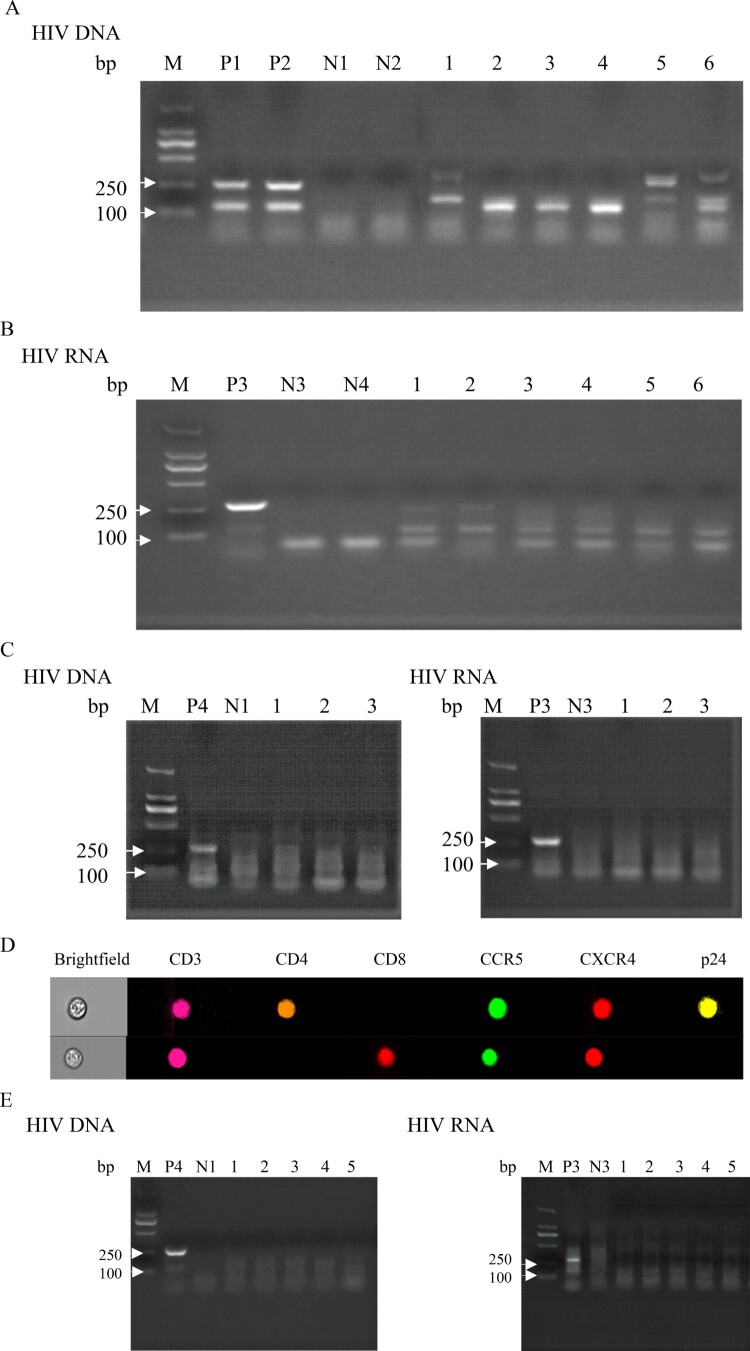
**Co-culture of HIV nucleic acid detected in purified spermatozoa from HIV-infected men with PBMCs of healthy men.** Purified spermatozoa from the TN group co-cultured with CD8-deleted PBMCs from healthy men. (A) HIV DNA detected from co-cultured cells and (B) HIV RNA extracted from the co-culture supernatant reverse transcribed into cDNA and used for nested PCR. (C) HIV DNA and RNA extracted from spermatozoa of three untreated HIV-infected men and cultured alone. (D) HIV-infected CD4^+^ T co-cultured with purified spermatozoon from untreated HIV infections. (E) HIV DNA and RNA extracted from purified spermatozoon of HIV-infections under ART co-cultured with CD8-deleted PBMCs of healthy men. Positive results valued as one of the 122 base pair and 264 base pair target bands positive; negative result was valued otherwise. M, DNA marker; P1, P2 and P4, DNA extracted from PBMCs of untreated HIV-infected men as positive controls; P3, HIV RNA extracted from the plasma of untreated HIV-infected men as positive controls. N1–N2, DNA extracted from PBMCs of healthy men as negative controls; N3–N4, HIV RNA extracted from plasma of healthy men as negative controls. Figure A: 1, 3, 5, HIV DNA extracted from co-cultured PBMCs of healthy men with purified spermatozoa of HIV infections; 2, 4, 6, HIV DNA extracted from co-cultured CD8-deleted-PBMCs of healthy men with purified spermatozoa of HIV infections. Figure B: 1–6, HIV RNA extracted from the co-cultured supernatant reverse transcribed into cDNA used for nested PCR (1-6 consistent with the order in Figure A). Figure C: 1–3, HIV DNA and RNA extracted from purified spermatozoon from untreated HIV infections cultured alone. Figure E: 1–5, HIV DNA and RNA extracted from purified spermatozoon from HIV infections under ART co-cultured with CD8-deleted-PBMCs of healthy men.

Whether spermatozoon carrying HIV had the ability to infect immune cells had important clinical implications; thus, we performed co-culture experiments co-culturing purified spermatozoon from HIV infections in the TN and ART groups with PBMCs from healthy men. The results of purified spermatozoon from the TN group showed that both HIV DNA extracted from co-cultured cells (Figure 5A) and HIV RNA from co-culture supernatants (Figure 5B) were positive. No positive signal was detected in the spermatozoa of the three untreated HIV infections cultured alone (Figure 5C). Moreover, imaging flow cytometry of co-cultured cells revealed that HIV carried by spermatozoa from the TN group could infect CD4^+^ T cells of healthy men (Figure 5D). Purified spermatozoon from the ART group showed that both HIV DNA extracted from co-cultured cells and HIV RNA from co-culture supernatants were negative (Figure 5E). These results provide strong evidence that HIV carried by spermatozoa can infect PBMCs from healthy men.

## Discussion

In this study, the distribution of HIV-positive spermatozoon and CD4^+^ T lymphocytes in semen was reported. Understanding whether HIV could infect spermatozoa could be beneficial to further elucidate the distribution and mechanism of HIV-infected cells in semen. Consistent with previous reports [11–13, 26, 27], our results demonstrated that HIV RNA and HIV p24 protein were detected in the spermatozoa of HIV-infected men. However, this was probably owing to different patient characteristics or limitations of DNA extraction technology wherein HIV DNA and proviral DNA were not extracted from the spermatozoa [13]. The low detection rates with RNAscope could be explained by the limitations of the RNA probes, which cannot contain all genotype sequences, or that some HIV adhere to the surface of the spermatozoa [6–8]. The HIV p24 antibodies have low specificity [28, 29]; thus, we stained semen cells and PBMCs from healthy men, and no obvious p24 cluster cells were found in the healthy group. The genome in the spermatozoa head is helical and transcriptionally silent [30, 31], and our results with RNAscope and previous studies with electron microscopy both revealed that HIV existed in the mitochondria of the spermatozoa neck region but not in the nucleus of the head [9, 10]. We detected HIV RNA in the neck of the spermatozoon and not in the head where the nucleus is present. If HIV integrates into the sperm genome, every cell in the newborn carries a segment of the HIV gene that is easy to detect. However, there are no case reports of seropositive children of serodiscordant couples. Therefore, we presumed that HIV may be derived from viral particles in the spermatozoa rather than being integrated with the spermatozoa genome of HIV infections.

Owing to the different concentrations of the medium and semen, and spermatozoa has the ability to swim from the lower semen layers to the upper medium. Therefore, it could be guaranteed that the isolated cells using the “swim-up” method were all motile spermatozoon. Under these circumstances, there are only spermatozoa in the co-culture system with no free virus from the semen. Our infectious experiments further demonstrated that HIV-positive spermatozoa have the ability to infect PBMCs from healthy men. In fact, almost all spermatozoa died around 3 days of co-culture, and HIV could not survive in the dead spermatozoon. We cultured for a total of 7 days; thus, viruses in the supernatant could only be produced by HIV-infected PBMCs in the co-culture system, and imaging flow experiment of co-cultured cells provide solid evidence of it.

Although HIV was undetectable in the peripheral blood after long durations of ART, we found that HIV p24-positive rates of spermatozoa significantly reduced but still present, indicating both the effectiveness of ART and the existence of viral compartmentalization [32–34]. Meanwhile, we found that the correlation of p24 positive rates of spermatozoa with baseline peripheral blood HIV RNA and CD4 in untreated patients suggested that the proportion of sperms carrying HIV may reflect disease progression. Whether the p24 positive rates of spermatozoa could further indicate the effect of antiviral therapy remains to be studied.

Moreover, we detected no HIV-positive spermatozoa in the ART group using RNAscope, probably owing to the limited number of cells in the semen smear and low HIV-positive rates after ART. The latency of HIV in spermatozoa warrants further study. Some studies assumed that HIV did not exist in the spermatozoa after long-term ART because HIV-positive partners under successful ART had limited transmission risk during unprotected sexual intercourse [35, 36]. Nevertheless, the transmission risk is strongly associated with plasma viral load, and the natural transmission rate of HIV without ART is approximately 1/30–1/200 per coital act rather than 100% [37]. Therefore, “U = U” did not imply no HIV-positive spermatozoa in the semen.

Receptor CD4 and co-receptors CCR5 and CXCR4 play important roles in HIV entry into target cells [15]. We found that the spermatozoa had low CD4 levels and that HIV-positive spermatozoon were mostly CD4 negative. Although the mechanism for HIV entry into these CD4-negative spermatozoon *in vivo* remains obscure, it may rely on cell-to-cell infection [38]. Previous studies reported that the testis is permissive to HIV or simian immunodeficiency virus infection [39, 40]. Moreover, TGCs can be infected in cell-associated pathways; thus, it could be hypothesized that during spermatogenesis, HIV can be transferred from HIV-infected lymphocytes or macrophages to spermatogonia through tight connections or virological synapses [14, 41]. Virological synapse infection requires stable cell-to-cell adhesion and interaction between infected T cells and non-immune cells, such as renal epithelial cells [42, 43]. Additionally, HIV-infected lymphocytes can transmit the virus to astrocytes in a CXCR4-dependent and CD4-independent manner [38]. Dominique Mahé reported that human TGCs lacked the HIV receptor CD4 [14]. However, alternative receptor galactoceramides or sulfogalactoceramides on the spermatozoa surface have been reported [44], and sperm-specific glycolipids could also serve as alternative receptors for gp120 [44]. Human mannose receptor protein on spermatozoa can also mediate HIV entry into it [45]. These results were obtained from *in vitro* experiments; therefore, further research is needed to validate whether HIV-infected spermatozoa *in vivo* use these pathways for entry. Moreover, using immunofluorescence some studies found that spermatozoa express CCR5 mRNA and CCR5 positive signals on the spermatozoa head [46–48]. We also found that CXCR4 or CCR5 levels of p24^+^ spermatozoa were higher than that those of p24^-^ spermatozoa. HIV co-receptors seem to play distinctive roles in HIV-infection of the spermatozoon, which may further promote the infection.

The proportion of spermatozoa in semen is much higher than that of CD4^+^ T cells; thus, although the HIV p24 positive rates of CD4^+^ T cells in semen are significantly higher than that of spermatozoa, most p24^+^ cells in semen are still spermatozoon. Additionally, the p24-positive rate of CD4^+^ T lymphocytes in semen was significantly higher than that of CD4^+^ T lymphocytes in blood, and the reasons for this may be as follows: (1) the reproductive system has an immune-privileged environment and HIV can avoid the scavenging effect of other immune cells to some degree [49]; (2) poor drug penetration into the reproductive system due to the blood-testis and blood-epididymal barriers [50]; and (3) clonal replication and compartmentalization of HIV in the male genital tract [51, 52]. We also found that the p24 positive rates of CD4 ^+ ^T cells in semen were significantly higher than those in spermatozoon, proving that HIV in semen was more likely to infect CD4 ^+ ^T cells. The above results suggest that viral clearance should be considered in both the peripheral blood and reproductive system when evaluating the therapeutic effect of ART. Serodiscordant couples should adhere to biomedical interventions and condom-protected intercourse to prevent sexual transmission of HIV, even infections, under long-term effective ART. However, whether HIV carried by spermatozoa can transfer into germ cells remains unclear [53].

This study has some limitations. First, we only stained and calculated spermatozoa and CD4+ T cells in semen, excluding epithelial cells and other immune cells. Second, the p24-positive rates of CD4+ T cells and spermatozoon, and expression levels of CD4 and co-receptors in semen were calculated by counting, which may not be fully representative of all actual situations. Third, this study had a limited sample size.

In conclusion, spermatozoa may be carriers of HIV. We described the distribution of spermatozoa and CD4^+^ T lymphocytes in semen and found that most of HIV-positive semen cells are spermatozoon. Spermatozoon from HIV-infected patients showed a predominant CD4-negative phenotype; moreover, we confirmed that HIV-positive spermatozoa could infect PBMCs through co-culture *in vitro*. The mechanism underlying HIV-infected spermatozoa in semen warrants further study.

## Supplementary Material

Supplemental Material

Supplemental Material

## References

[1] Liu J, Hou Y, Sun L, et al. High population-attributable fractions of traditional risk factors for non-AIDS-defining diseases among people living with HIV in China: a cohort study. Emerg Microbes Infect. 2021;10(1):416–423. doi:10.1080/22221751.2021.1894904. 33620297 PMC7971336

[2] Liu J, Wang L, Hou Y, et al. Immune restoration in HIV-1-infected patients after 12 years of antiretroviral therapy: a real-world observational study. Emerg Microbes Infect. 2020;9(1):2550–2561. doi:10.1080/22221751.2020.1840928. 33131455 PMC7733958

[3] Zagury D, Bernard J, Leibowitch J, et al. HTLV-III in cells cultured from semen of two patients with AIDS. Science. 1984;226(4673):449–451. doi: doi:10.1126/science.6208607. 6208607

[4] Houzet L, Matusali G, Dejucq-Rainsford N. Origins of HIV-infected leukocytes and virions in semen. J Infect Dis. 2014;210(3):S622–S630. doi:10.1093/infdis/jiu328. 25414416

[5] Anderson DJ, Politch JA, Nadolski AM, et al. Targeting trojan horse leukocytes for HIV prevention. AIDS. 2010;24(2):163–187. doi:10.1097/QAD.0b013e32833424c8. 20010071 PMC4097178

[6] Bujan L, Hollander L, Coudert M, et al. Safety and efficacy of sperm washing in HIV-1-serodiscordant couples where the male is infected: results from the European CREAThE network. AIDS. 2007;21(14):1909–1914. doi:10.1097/QAD.0b013e3282703879. 17721098

[7] Eke AC, Oragwu C. Sperm washing to prevent HIV transmission from HIV-infected men but allowing conception in sero-discordant couples. Cochrane Database Syst Rev. 2011;19(1):CD008498. doi:10.1002/14651858. 21249711

[8] Garrido N, Meseguer M, Bellver J, et al. Report of the results of a 2 year programme of sperm wash and ICSI treatment for human immunodeficiency virus and hepatitis C virus serodiscordant couples. Hum Reprod. 2004;19(11):2581–2586. doi:10.1093/humrep/deh460. 15319386

[9] Scofield VL, Rao B, Broder S, et al. Hiv interaction with sperm. AIDS. 1994;8(12):1733–1736. doi:10.1097/00002030-199412000-00018. 7888126

[10] Dussaix E, Guetard D, Dauguet C, et al. Spermatozoa as potential carriers of HIV. Res Virol. 1993;144(6):487–495. doi:10.1016/S0923-2516(06)80064-6. 8140292

[11] Ceballos A, Remes Lenicov F, Sabatte J, et al. Spermatozoa capture HIV-1 through heparan sulfate and efficiently transmit the virus to dendritic cells. J Exp Med. 2009;206(12):2717–2733. doi:10.1084/jem.20091579. 19858326 PMC2806607

[12] Muciaccia B, Corallini S, Vicini E, et al. HIV-1 viral DNA is present in ejaculated abnormal spermatozoa of seropositive subjects. Hum Reprod. 2007;22(11):2868–2878. doi:10.1093/humrep/dem288. 17855413

[13] Dulioust E, Tachet A, De Almeida M, et al. Detection of HIV-1 in seminal plasma and seminal cells of HIV-1 seropositive men. J Reprod Immunol. 1998;41(1-2):27–40. doi:10.1016/S0165-0378(98)00047-3. 10213299

[14] Mahe D, Matusali G, Deleage C, et al. Potential for virus endogenization in humans through testicular Germ cell infection: the case of HIV. J Virol. 2020;94(24). doi:10.1128/JVI.01145-20. PMC7925188 32999017

[15] Chen B. Molecular mechanism of HIV-1 entry. Trends Microbiol. 2019;27(10):878–891. doi:10.1016/j.tim.2019.06.002. 31262533 PMC6744290

[16] Vidricaire G, Gauthier S, Tremblay MJ. HIV-1 infection of trophoblasts Is independent of gp120/CD4 interactions but relies on heparan sulfate proteoglycans. J Infect Dis. 2007;195(10):1461–1471. doi:10.1086/515576. 17436226

[17] Byrn RA, Zhang D, Eyre R, et al. HIV-1 in semen: an isolated virus reservoir. The Lancet. 1997;350(9085):1141. doi:10.1016/S0140-6736(97)24042-0. 9343504

[18] McGrath KE, Catherman SC, Palis J. Delineating stages of erythropoiesis using imaging flow cytometry. Methods. 2017;112:68–74. doi:10.1016/j.ymeth.2016.08.012. 27582124

[19] Fukazawa Y, Lum R, Okoye AA, et al. B cell follicle sanctuary permits persistent productive simian immunodeficiency virus infection in elite controllers. Nat Med. 2015;21(2):132–139. doi:10.1038/nm.3781. 25599132 PMC4320022

[20] Micci L, Alvarez X, Iriele RI, et al. Cd4 depletion in SIV-infected macaques results in macrophage and microglia infection with rapid turnover of infected cells. PLoS Pathog. 2014;10(10):e1004467. doi:10.1371/journal.ppat.1004467. 25356757 PMC4214815

[21] Smedley J, Turkbey B, Bernardo ML, et al. Tracking the luminal exposure and lymphatic drainage pathways of intravaginal and intrarectal inocula used in nonhuman primate models of HIV transmission. PLoS One. 2014;9(3):e92830. doi:10.1371/journal.pone.0092830. 24667371 PMC3965472

[22] Mujugira A, Coombs RW, Heffron R, et al. Seminal HIV-1 RNA detection in heterosexual african Men initiating antiretroviral therapy. J Infect Dis. 2016;214(2):212–215. doi:10.1093/infdis/jiw131. 27053765 PMC4918825

[23] Palich R, Ghosn J, Chaillon A, et al. Viral rebound in semen after antiretroviral treatment interruption in an HIV therapeutic vaccine double-blind trial. AIDS. 2019;33(2):279–284. doi:10.1097/QAD.0000000000002058. 30325777

[24] Song JW, Zhang C, Fan X, et al. Immunological and inflammatory profiles in mild and severe cases of COVID-19. Nat Commun. 2020;11(1):3410. doi:10.1038/s41467-020-17240-2. 32641700 PMC7343781

[25] Lamers SL, Rose R, Ndhlovu LC, et al. The meningeal lymphatic system: a route for HIV brain migration? J Neurovirol. 2016;22(3):275–281. doi:10.1007/s13365-015-0399-y. 26572785 PMC4868798

[26] Wang D, Li LB, Hou ZW, et al. The integrated HIV-1 provirus in patient sperm chromosome and its transfer into the early embryo by fertilization. PLoS One. 2011;6(12):e28586. doi:10.1371/journal.pone.0028586. 22194862 PMC3237474

[27] Bagasra O, Farzadegan H, Seshamma T, et al. Detection of HIV-1 proviral DNA in sperm from HIV-1-infected men. AIDS. 1994;8(12):1669–1674. doi:10.1097/00002030-199412000-00005. 7888115

[28] Pardons M, Baxter AE, Massanella M, et al. Single-cell characterization and quantification of translation-competent viral reservoirs in treated and untreated HIV infection. PLoS Pathog. 2019;15(2):e1007619. doi:10.1371/journal.ppat.1007619. 30811499 PMC6411230

[29] Gray ER, Bain R, Varsaneux O, et al. P24 revisited. AIDS. 2018;32(15):2089–2102. doi:10.1097/QAD.0000000000001982. 30102659 PMC6139023

[30] Zhang Y, Shi J, Rassoulzadegan M, et al. Sperm RNA code programmes the metabolic health of offspring. Nature Reviews Endocrinology. 2019;15(8):489–498. doi:10.1038/s41574-019-0226-2. PMC6626572 31235802

[31] Lishko PV, Botchkina IL, Kirichok Y. Progesterone activates the principal Ca2+ channel of human sperm. Nature. 2011;471(7338):387–391. doi:10.1038/nature09767. 21412339

[32] Browne FA, Wechsberg WM. The intersecting risks of substance use and HIV risk among substance-using South African men and women. Curr Opin Psychiatry. 2010;23(3):205–209. doi:10.1097/YCO.0b013e32833864eb. 20308902 PMC3784346

[33] Chaillon A, Smith DM, Vanpouille C, et al. HIV trafficking between blood and semen during early untreated HIV infection. JAIDS J Acquir Immune Defic Syndr. 2017;74(1):95–102. doi:10.1097/QAI.0000000000001156. 27548440 PMC5140710

[34] Chaillon A, Gianella S, Wertheim JO, et al. Hiv migration between blood and cerebrospinal fluid or semen over time. J Infect Dis. 2014;209(10):1642–1652. doi:10.1093/infdis/jit678. 24302756 PMC3997580

[35] Cook R. Antiretroviral treatment can reduce the risk of HIV transmission between male partners to ‘zero’. Br Med J. 2019;366:l4915. doi:10.1136/bmj.l4915. 31455630

[36] Hassan MF, Mahmood S, Dhamija B, et al. An association between cerebral aneurysm re-bleed and CT angiography – more than a coincidence? Br J Neurosurg. 2011;25(6):734–735. doi:10.3109/02688697.2011.584984. 21767130

[37] Rekart ML, Macintosh J. Acute primary HIV infection. Can Med Assoc J. 2011;183(11):1280. doi:10.1503/cmaj.101605. 21646465 PMC3153518

[38] Li GH, Anderson C, Jaeger L, et al. Cell-to-cell contact facilitates HIV transmission from lymphocytes to astrocytes via CXCR4. AIDS. 2015;29(7):755–766. doi:10.1097/QAD.0000000000000605. 25985398 PMC4438861

[39] Roulet V, Satie AP, Ruffault A, et al. Susceptibility of human testis to human immunodeficiency virus-1 infection in situ and in vitro. Am J Pathol. 2006;169(6):2094–2103. doi:10.2353/ajpath.2006.060191. 17148672 PMC1762481

[40] Le Tortorec A, Le Grand R, Denis H, et al. Infection of semen-producing organs by SIV during the acute and chronic stages of the disease. PLoS One. 2008;3(3):e1792. doi:10.1371/journal.pone.0001792. 18347738 PMC2268241

[41] Hubner W, McNerney GP, Chen P, et al. Quantitative 3D video microscopy of HIV transfer across T cell virological synapses. Science. 2009;323(5922):1743–1747. doi:10.1126/science.1167525. 19325119 PMC2756521

[42] Chen P, Chen BK, Mosoian A, et al. Virological synapses allow HIV-1 uptake and gene expression in renal tubular epithelial cells. J Am Soc Nephrol. 2011;22(3):496–507. doi:10.1681/ASN.2010040379. 21335514 PMC3060443

[43] Hughes K, Akturk G, Gnjatic S, et al. Proliferation of HIV-infected renal epithelial cells following virus acquisition from infected macrophages. AIDS. 2020;34(11):1581–1591. doi:10.1097/QAD.0000000000002589. 32701578 PMC7579771

[44] Gadella BM, Hammache D, Pieroni G, et al. Glycolipids as potential binding sites for HIV: topology in the sperm plasma membrane in relation to the regulation of membrane fusion. J Reprod Immunol. 1998;41(1-2):233–253. doi:10.1016/S0165-0378(98)00061-8. 10213313

[45] Fanibunda SE, Velhal SM, Raghavan VP, et al. Cd4 independent binding of HIV gp120 to mannose receptor on human spermatozoa. JAIDS J Acquir Immune Defic Syndr. 2008;48(4):389–397. doi:10.1097/QAI.0b013e318179a0fb. 18614929

[46] Isobe T, Minoura H, Tanaka K, et al. The effect of RANTES on human sperm chemotaxis. Hum Reprod. 2002;17(6):1441–1446. doi:10.1093/humrep/17.6.1441. 12042258

[47] Muciaccia B, Padula F, Gandini L, et al. HIV-1 chemokine co-receptor CCR5 is expressed on the surface of human spermatozoa. AIDS. 2005;19(13):1424–1426. doi:10.1097/01.aids.0000180809.04427.04. 16103775

[48] Muciaccia B, Padula F, Vicini E, et al. Beta-chemokine receptors 5 and 3 are expressed on the head region of human spermatozoon. FASEB J. 2005;19(14):2048–2050. doi:10.1096/fj.05-3962fje. 16174786

[49] Le Tortorec A, Matusali G, Mahe D, et al. From ancient to emerging infections: The odyssey of viruses in the male genital tract. Physiol Rev. 2020;100(3):1349–1414. doi:10.1152/physrev.00021.2019. 32031468

[50] Matusali G, Dereuddre-Bosquet N, Le Tortorec A, et al. Detection of simian immunodeficiency virus in semen, urethra, and male reproductive organs during efficient highly active antiretroviral therapy. J Virol. 2015;89(11):5772–5787. doi:10.1128/JVI.03628-14. 25833047 PMC4442442

[51] Kariuki SM, Selhorst P, Anthony C, et al. Compartmentalization and clonal amplification of HIV-1 in the male genital tract characterized using next-generation sequencing. J Virol. 2020;94(12). doi:10.1128/JVI.00229-20. PMC7307092 32269124

[52] Council OD, Zhou S, McCann CD, et al. Deep sequencing reveals compartmentalized HIV-1 in the semen of Men with and without sexually transmitted infection-associated urethritis. J Virol. 2020;94(12). doi:10.1128/JVI.00151-20. PMC7307086 32269129

[53] Zhou Z, Ma P, Feng Y, et al. The inference of HIV-1 transmission direction between a man who has sex with men and his heterosexual wife based on the sequences of HIV-1 quasi-species. Emerg Microbes Infect. 2021;10(1):1209–1216. doi:10.1080/22221751.2021.1938693. 34077305 PMC8676586

