# Supplementary material for: Characterization and distribution of HIV-infected cells in semen
Source: Emerg Microbes Infect. 2022 Mar 21;11(1):860–72. doi: 10.1080/22221751.2022.2049982 (PMC8942556; doi:10.1080/22221751.2022.2049982)
Supplement: Supplemental Material [file TEMI_A_2049982_SM0667.docx]

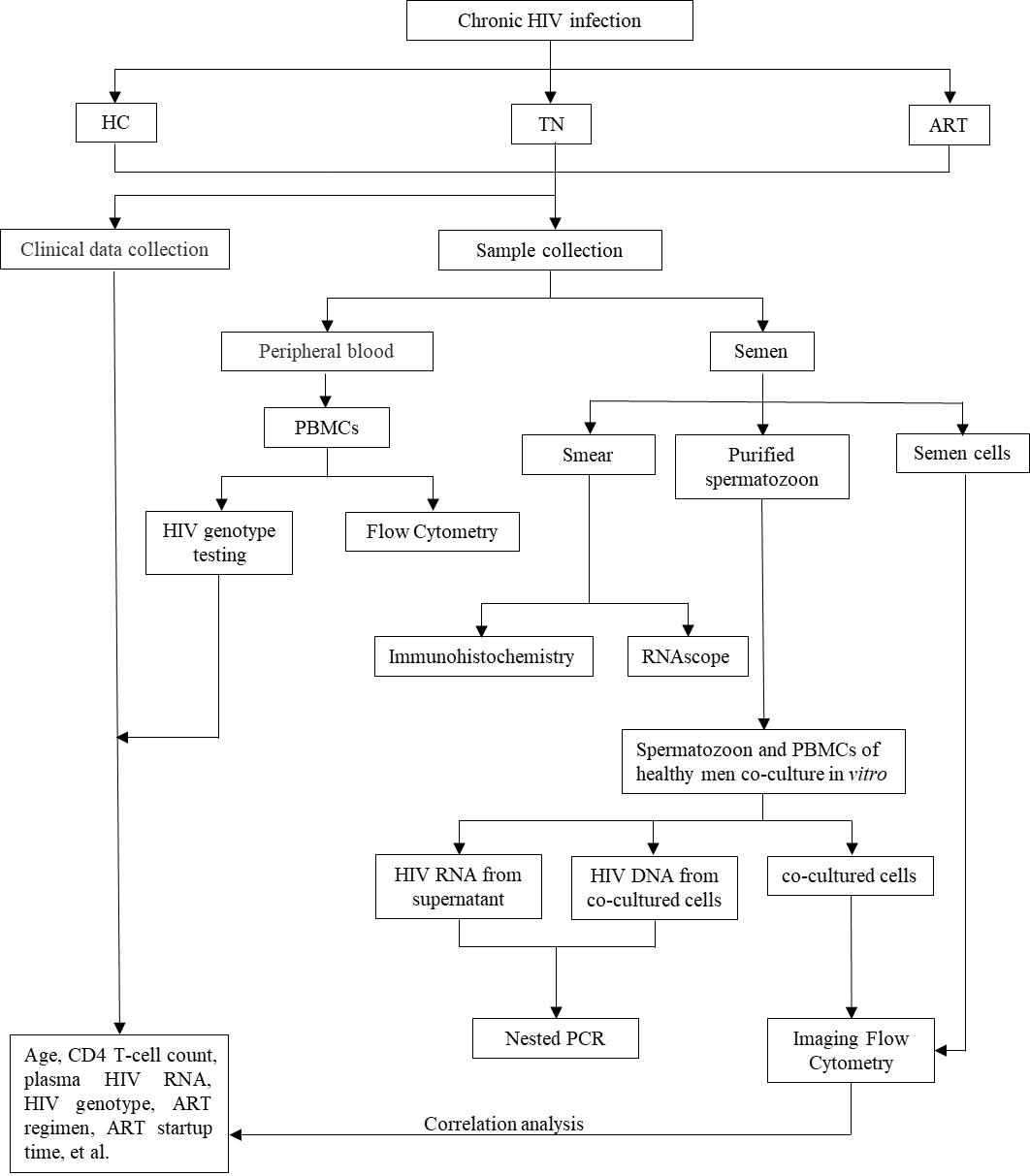


Supplementary Figure 1. Flowchart of study design.


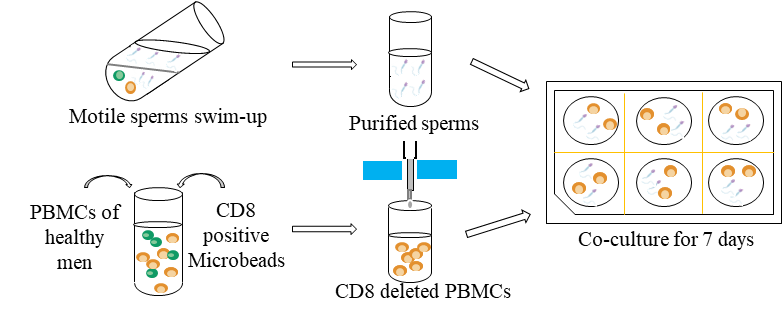


Supplementary Figure 2. Flowchart for co-culture experiments after purified spermatozoon by “swim-up” method.

**
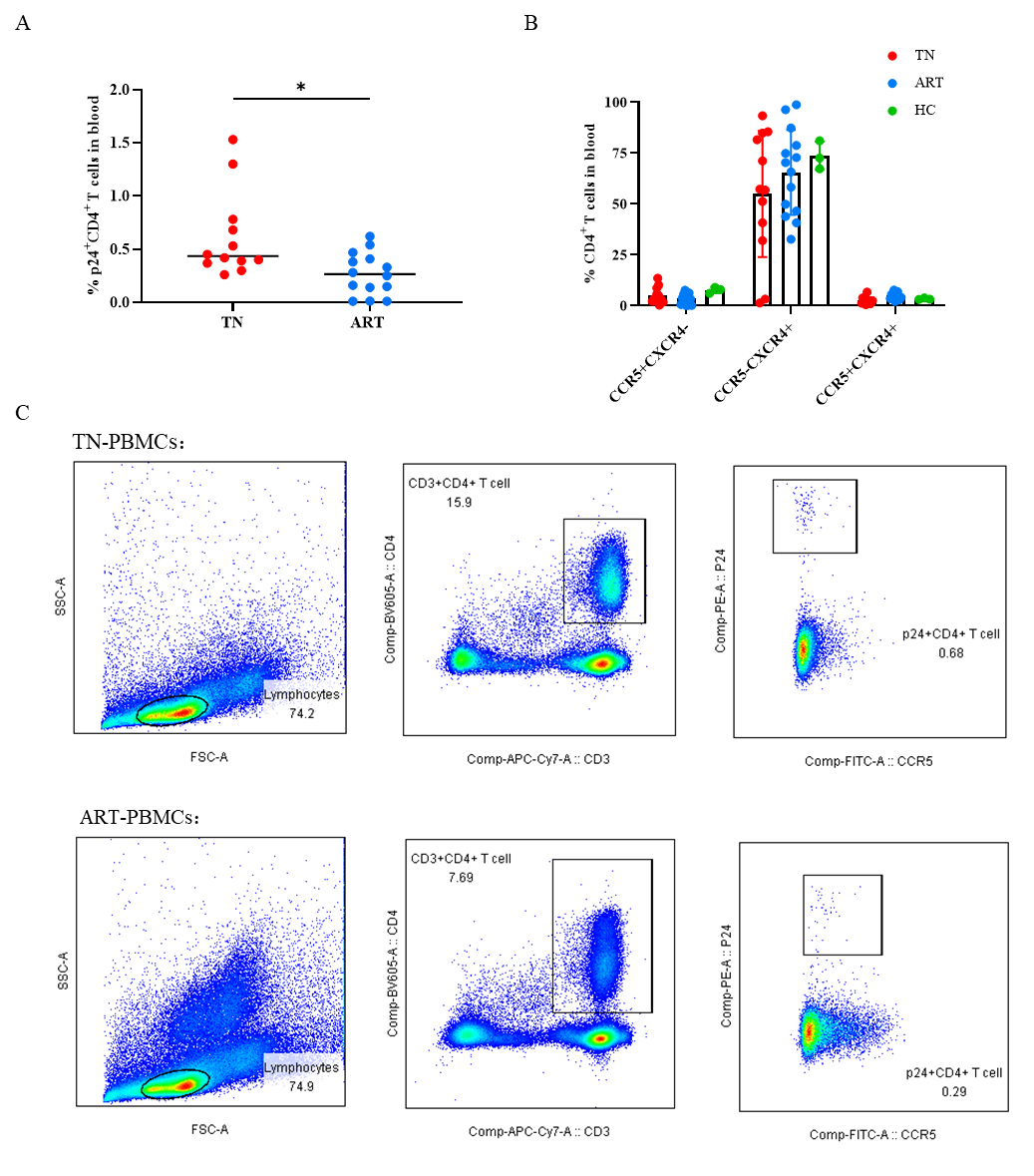
**

Supplementary Figure 3. HIV p24 protein, CCR5, and CXCR4 levels of CD4^+^ T lymphocytes in the peripheral blood. (A) p24 positive rates of CD4^+^ T lymphocytes in peripheral blood from TN and ART groups. (B) Co-receptors R5 and X4 expression on CD4^+^ T lymphocytes in peripheral blood of HIV-infected men and healthy men. (C) Typical flow diagram of p24^+^ CD4^+^ T cells in peripheral blood of HIV-infected men in the TN and ART group.
