# Supplementary material for: Characterization and distribution of HIV-infected cells in semen
Source: Emerg Microbes Infect. 2022 Mar 21;11(1):860–72. doi: 10.1080/22221751.2022.2049982 (PMC8942556; doi:10.1080/22221751.2022.2049982)
Supplement: Supplemental Material [file TEMI_A_2049982_SM0655.docx]

Supplementary Table 1. Primer sequence of nested PCR

| Name | Site | 5’-3’ | Gene | Length |
| --- | --- | --- | --- | --- |
| outerA | 5783-5804 | TTGGGTGTCAACATAGCAGAATAGG  (SEQ ID NO.1) | vpr | 446bp |
|  | 6228-6210 | CTCTCATTGCCACTGTCTTCTG  (SEQ ID NO.2) | vpu |  |
| innerA | 5958-5978 | GCTTAGGCATCTCTATGGCAGGA  (SEQ ID NO.3)  GCTTAGGCATTCCTATGGCAGGA  (SEQ ID NO.4) | tat | 264bp |
|  | 6221-6203 | TGCCACTGTCTTCTGCTCTTTC  (SEQ ID NO.5) | vpu |  |
| outerB | 1235-1256 | CACCTAGAACTTTAAATGCATGGGT  (SEQ ID NO.6) | gag | 778bp |
|  | 2012-1992 | CTAGGGGCCCTGCAATTTTGGCT  (SEQ ID NO.7)  CTAGGGGCCCTGCAATTCTGGCT  (SEQ ID NO.8) | gag |  |
| innerB | 2012-1992 | CATTCTGGACATAAACAAGGCC  (SEQ ID NO.9)  CATTCTGGACATGAACAAGACC  (SEQ ID NO.10) | gag | 122bp |
|  | 1756-1737 | GGACCAACAAGGTTTCTGTCATC  (SEQ ID NO.11) | gag |  |

Supplementary Table 2. Nested PCR reaction system

| Name | The first round of PCR | The second round of PCR |
| --- | --- | --- |
| 2*Mix | 12.5 uL | 12.5 uL |
| Primer | outer A 0.5 uL | innerA 0.5 uL |
|  | outerA 0.5 uL | innerA 0.5 uL |
|  | outerB 0.5 uL | innerB 0.5 uL |
|  | outerB 0.5 uL | innerB 0.5 uL |
| H_2_O | 9.5 uL | 9.5 uL |
| DNA | 1 uL | 1 uL |
| Total volume | 25 uL | 25 uL |

Supplementary Table 3. Nested PCR reaction conditions

| Name | Temperature | The first round of PCR | The second round of PCR |
| --- | --- | --- | --- |
| Pre-denaturation | 94 ℃ | 3 min | 3 min |
| Denaturation | 94 ℃ | 30 s | 30 s |
| Renaturation | 52 ℃ | 30 s | 30 s |
| Extension | 72 ℃ | 90 s | 30 s |
| Post-extension | 72 ℃ | 5min | 5min |

Supplementary Table 4. Histograms of fluorescent intensity of p24 antigens by the flow cytometry analysis

| Number | Intensity of p24 | Number | Intensity of p24 |
| --- | --- | --- | --- |
| 1 | 1978.47 | 5 | 8368.51 |
| 2 | 33.05 | 6 | 11.62 |
| 3 | 1798.41 | 7 | 7563.29 |
| 4 | 956.22 | 8 | 2918.49 |
